# Technical Evaluation of Commercial Mutation Analysis Platforms and Reference Materials for Liquid Biopsy Profiling

**DOI:** 10.3390/cancers12061588

**Published:** 2020-06-16

**Authors:** Sabrina Weber, Benjamin Spiegl, Samantha O. Perakis, Christine M. Ulz, Peter M. Abuja, Karl Kashofer, Paul van der Leest, Maria Aguirre Azpurua, Menno Tamminga, Dan Brudzewsky, Dominic G. Rothwell, Sumitra Mohan, Alexander Sartori, Rita Lampignano, Yves Konigshofer, Markus Sprenger-Haussels, Harriet Wikman, Inger R. Bergheim, Vera Kloten, Ed Schuuring, Michael R. Speicher, Ellen Heitzer

**Affiliations:** 1 Institute of Human Genetics, Diagnostic & Research Center for Molecular BioMedicine, Medical University of Graz, 8010 Graz, Austria; sabrina.weber@medunigraz.at (S.W.); benajmin.spiegl@medunigraz.at (B.S.); samantha.perakis@medunigraz.at (S.O.P.); michael.speicher@medunigraz.at (M.R.S.); 2 Christian Doppler Laboratory for Liquid Biopsies for Early Detection of Cancer, Medical University of Graz, 8010 Graz, Austria; peter.abuja@medunigraz.at; 3 Institute of Pathology, Diagnostic & Research Center for Molecular BioMedicine, Medical University of Graz, 8010 Graz, Austria; christine.ulz@medunigraz.at (C.M.U.); karl.kashofer@medunigraz.at (K.K.); p.van.der.leest@umcg.nl (P.v.d.L.); 4 University of Groningen, University Medical Center of Groningen, 9713 GZ Groningen, The Netherlands; ma_aguirre1@yahoo.com.ar (M.A.A.); m.tamminga@umcg.nl (M.T.); e.schuuring@umcg.nl (E.S.); 5 LGC SeraCare Life Sciences, Milford, MA 01757, USA; dan.brud@gmail.com (D.B.); Yves.Konigshofer@lgcgroup.com (Y.K.); 6 Cancer Research UK MI, University of Manchester, Manchester SK10 4TG, UK; Dominic.Rothwell@manchester.ac.uk (D.G.R.); sumitra.mohan@manchester.ac.uk (S.M.); 7 Agena Bioscience GmbH, 22761 Hamburg, Germany; Alexander.Sartori@agenabio.com; 8 Bayer AG, Biomarker Research, 42113 Wuppertal, Germany; rital@miltenyibiotec.de (R.L.); vera.kloten@bayer.com (V.K.); 9 QIAGEN GmbH, 40724 Hilden, Germany; Markus.Sprenger-Haussels@qiagen.com; 10 University Medical Center Hamburg-Eppendorf, 20251 Hamburg, Germany; h.wikman@uke.de; 11 Department of Cancer Genetics, Institute of Cancer Research, Oslo University Hospital, N-0310 Oslo, Norway; Inger.Riise.Bergheim@rr-research.no

**Keywords:** ctDNA, circulating tumor DNA, cfDNA, cell-free DNA, molecular profiling, mutation analysis, reference material, diagnostic leukaphereses, assay validation, performance assessment

## Abstract

Molecular profiling from liquid biopsy, in particular cell-free DNA (cfDNA), represents an attractive alternative to tissue biopsies for the detection of actionable targets and tumor monitoring. In addition to PCR-based assays, Next Generation Sequencing (NGS)-based cfDNA assays are now commercially available and are being increasingly adopted in clinical practice. However, the validity of these products as well as the clinical utility of cfDNA in the management of patients with cancer has yet to be proven. Within framework of the Innovative Medicines Initiative (IMI) program CANCER-ID we evaluated the use of commercially available reference materials designed for ctDNA testing and cfDNA derived from Diagnostic Leukaphereses (DLA) for inter- and intra-assay as well as intra- and inter-laboratory comparisons. In three experimental setups, a broad range of assays including ddPCR, MassARRAY and various NGS-based assays were tested. We demonstrate that both reference materials with predetermined VAFs and DLA samples are extremely useful for the performance assessment of mutation analysis platforms. Moreover, our data indicate a substantial variability of NGS assays with respect to sensitivity and specificity highlighting the importance of extensive validation of the test performance before offering these tests in clinical routine practice.

## 1. Introduction

Screening for actionable or therapy resistance-associated mutations has the potential to improve outcomes for cancer patients and has already entered clinical practice [1,2,3,4]. Tests for molecular profiling range from simple to complex and include various technologies such polymerase chain reaction (PCR)-based approaches, Sanger sequencing, pyrosequencing, multiplex ligation-dependent probe amplification (MLPA), or mass spectrometry (MS). As costs and turnaround time of Next Generation Sequencing (NGS) significantly decreased in the last ten years, and bioinformatics analyses, as well as the harmonization of knowledgebases to facilitate the clinical interpretation of genomic results improved, NGS is increasingly being used for comprehensive molecular profiling.

Comprehensive genetic testing for somatic alterations is usually performed on formalin-fixed, paraffin-embedded (FFPE) tissue. However, this archiving process can alter nucleic acids by chemical modification and can cause DNA cross-linking and fragmentation. This poses analytical challenges in terms of quality and quantity [5,6]. Moreover, intratumor heterogeneity adds another layer of complexity [7,8,9]. Finally, tumors are highly dynamic systems and may change under the selective pressure of prior therapies, which mandates consecutive sampling over time. Due to the invasive nature and the associated risks, acquisition of multiple tumor biopsies over time is limited. To avoid morbidity of traditional biopsies and enable more frequent monitoring, liquid biopsies are an attractive alternative in monitoring the evolution of tumor genomes during exposure to a broad range of treatments [10]. The analysis of circulating tumor DNA (ctDNA) has been demonstrated to better mirror the clinical situation by reflecting a composite mutational landscape [11,12,13]. Although, in most cases, tumor-derived DNA is just a small percentage of the total cell-free DNA (cfDNA) fraction in circulation [14,15,16], recent technological improvements, such as the introduction of unique molecular identifiers (UMI) to reduce sequencing noise, now enable the detection of rare variants down to 0.1% [17,18,19]. 

Applications for liquid biopsies are rapidly expanding and numerous research, industry, and commercial cfDNA-sequencing assays have been developed. In some centers, laboratory-developed tests (LDT) have been implemented. A prime example is the MSK-ACCESS assay, a comprehensive liquid biopsy test that involves deep sequencing of 129 key cancer-associated genes selected from the Memorial Sloan Kettering Cancer Center (MSK) solid tumor genomic-profiling assay, MSK-IMPACT™, the first tumor-profiling LDT that received clearance through the Food & Drug Administration (FDA) [20]. However, the development of such an assay is extremely complex, costly and requires time as well as highly specialized clinical laboratory expertise. Therefore, service providers like Guardant Health (Guardant 360, 73 genes), Foundation Medicine (FoundationOne Liquid, 70 genes) or Personal Genome Diagnostics (PLASMASELECT, 64 genes) offer centralized testing and are at present predominately being used in the US [21,22,23]. In addition, many biotechnology companies such as Illumina, QIAGEN, Roche, or Thermo Fisher focus on developing ctDNA analysis technologies or already have made workflows commercially available [21]. Yet, most of these kits have not been rigorously tested and studies demonstrating the consistency between these commercial products for the analysis of ctDNA are lacking [24,25]. 

**Figure 1:**
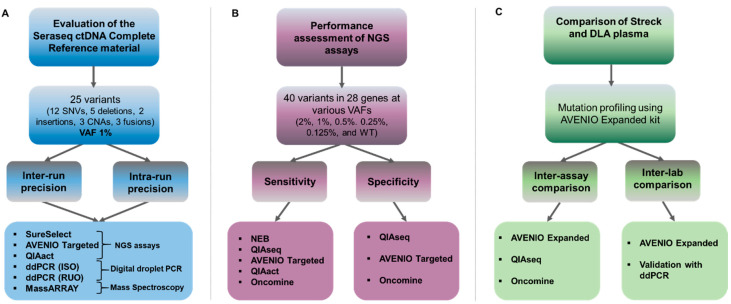
Experimental setup to assess commercially available reference material and mutation analysis kits. (**A**) Multicenter evaluation of the Seraseq ctDNA Complete reference material using three NGS panels (a custom SureSelect design [Agilent], the AVENIO Targeted kit [Roche], and the QIAact Lung UMI Panel [QIAGEN]), two digital droplet PCR assays (ddPCR) (one ISO15189 certified [ISO] and one research use only [RUO], and the MassARRAY (UltraSeek lung panel [Agena]). Intra-run precision was evaluated by testing the VAF1% reference material in triplicate in a single complete mutational analysis workflow. Inter-run reproducibility was assessed by testing the sample in three separate mutational analysis workflows. (**B**) Using the Seraseq ctDNA reference material v2, five commercially available mutation analysis assays targeting clinically relevant genes were tested with respect to sensitivity and specificity. (**C**) Assessment of Diagnostic LeukApheresis (DLA) for inter-assay comparison at a single site (MUG) and for inter-laboratory comparisons at two sites using the AVENIO Expanded kit (MUG and UMCG). A subset of variants was validated using ddPCR.

Here, we summarize data from three experimental setups to comprehensively assess mutation analysis platforms performed in combination with commercially available reference material and plasma from Diagnostic LeukAphereses (DLA) within the framework of the Innovative Medicines Initiative (IMI) program CANCER-ID (http://www.cancer-id.eu) (Figure 1). This consortium aims at the evaluation of technologies for blood-based biomarker analysis and establishment of criteria for benchmarking different technologies as well as comparative data on different methods for the molecular analysis of liquid biopsy components. 

## 2. Results

### 2.1. Assessment of the Seraseq ctDNA Complete Reference Material for Multicenter Evaluation of ctDNA Assays

In this part of our study, we assessed the use of a complex ctDNA reference material, i.e., the Seraseq ctDNA Complete reference material (VAF, variant allele frequency of 1%) for multicenter evaluation by five Cancer-ID partners (Figure 1A). This included three NGS panels: a customized hybrid capture SureSelect custom panel from Agilent (performed at University of Manchester—UNIMAN); the AVENIO Targeted Assay from Roche, a commercially available hybrid capture-based custom panel which employs UMIs; and a commercially available amplicon panel QIAact Lung UMI Panel from QIAGEN (both performed at Medical University of Graz—MUG). In addition, we evaluated two droplet digital PCR (ddPCR) assays (one research use only assay performed at Bayer and one ISO15189 validated assay performed at the University Medical Center Groningen—UMCG) and the UltraSeek lung panel (MassARRAY performed at Agena Bioscience). Since qPCR-based analysis can only interrogate single or few targets and the content of the various NGS panels varied, we focused our analyses on the most clinically relevant mutations, i.e., *BRAF* V600E, *EGFR* T790M, *EGFR* L858R, *KRAS* G12C and *KRAS* G12D. 

**Figure 2:**
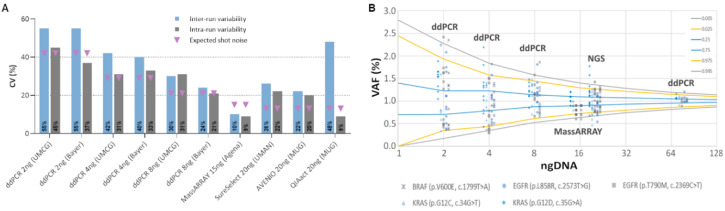
Inter- and intra-run variability of three qPCR-based and three NGS assays calculated from five clinically relevant mutations in three replicates. The use of the SeraCare Seraseq ctDNA Complete reference material (VAF1%) was evaluated using two digital droplet PCR assays (ddPCR) (one ISO15189 certified [ISO (UMCG)] and one research use only [RUO (Bayer)], the MassARRAY (UltraSeek lung panel) and three NGS panels (a custom SureSelect design, the AVENIO Targeted kit, and the QIAact Lung UMI Panel). Five clinically relevant mutations, i.e., *BRAF* V600E, *EGFR* T790M, *EGFR* L858R, *KRAS* G12C and *KRAS* G12D were taken into account. (**A**) Plotted are the coefficients of variations (CV) of observed VAFs of all tested assays. Shown are inter- and intra-run variability (three replicates each) as well as the calculated random error (shot noise) based on the availability of mutant input molecules. (**B**) Shown are VAFs for various input masses of DNA calculated from three NGS panels (20 ng), the MassARRAY (15 ng), and two ddPCR assays (8, 4, and 2 ng), demonstrating that the amount of input DNA (i.e., available molecules) clearly affects the variability of a test. Plotted are the respective VAFs as well as an inverse binomial distribution based on the trials (how many copies of DNA were analyzed), the likelihood of an event happening (0.01 for 1% VAF), and the location along the cumulative distribution function (colored line, e.g., 2.5th percentile would be 0.025). For example, when sampling a 4-ng input DNA, one can expect to observe a 1% variant between VAFs of 0.44% and 1.57% with a likelihood of 95%, or between 0.35% and 1.85% with a likelihood of and 99%.

These five variants were consistently detected across all platforms with an average VAF of 1.03% (range 0.5–1.7%) (Figure S1 and Table S1). The variability of intra-run variant calls (assessed by three replicates in the same run) was comparable between both ddPCR assays (input 8 ng each; Bayer CV 24%; UMCG CV 30%) and two of the three NGS assays (input 20 ng each; SureSelect CV 26%; AVENIO CV 22%) (Figure 2A). We observed the lowest and the highest variabilities for MassARRAY (input 15 ng, CV 19%) and the third NGS assay, QIAact (input 20 ng, CV 48%) (Figure 2A), respectively. As expected, the inter-run reproducibility—assessed by testing the same sample in three separate mutational analysis workflows—was slightly higher for all methods compared to intra-run variability (Table S1). 

One challenge in molecular profiling represents the limited amount of cfDNA available in most analyses, which is important to consider in the context of analytical sensitivity and precision. To this end, we explored the impact of the amount of DNA use on the variability of ddPCR assays. To assess how much of the variability found can be attributed to random errors associated with the availability of mutant fragments, we took the shot (Poisson) noise into account. Naturally, assays that analyze less input would be expected to have less precision around a VAF of 1% compared to assays with higher input (Figure 2A). Indeed, lowering the DNA input from 8 to 4 and 2 was clearly associated with a decrease of variant calling precision (Figure 2A,B and Figure S2). Compared to ddPCR, the variant call confidence was better for the MassARRAY and the NGS assays, which can be attributed to a higher input DNA of 15 and 20ng, respectively. The use of a 75-ng input for ddPCR resulted in the highest precision; however, in many clinical scenarios, insufficient plasma is unable to yield such high cfDNA amounts (Figure 2B). Overall, the measured variability was equal or slightly higher than the expected variability, except for the MassARRAY which turned out to be more precise than expected. 

### 2.2. Performance Assessment of Commercially Available Mutation Analysis Platforms

#### 2.2.1. Sensitivity Assessment of Five Commercially Available Mutation Assays Using the Seraseq^®^ ctDNA Reference Materials

**Figure 3:**
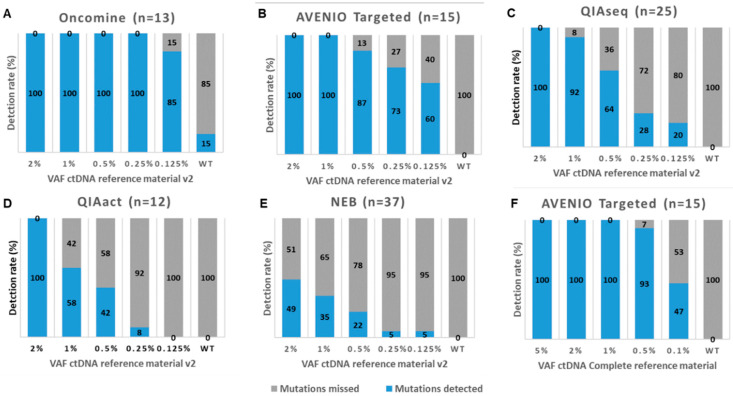
Detection rates of five commercially available mutation analysis kits. Shown are the detection rates of mutations (SNVs and indels) in the Seraseq ctDNA Reference Material v2 at various variant allele frequencies (VAF) for (**A**) the Oncomine Lung cfDNA Assay (Oncomine), (**B**) the AVENIO Targeted Kit (AVENIO Targeted), (**C**) the QIAseq Human Actionable Solid Tumor Panel (QIAseq), (**D**) the GeneRead QIAact Lung UMI Panel (QIAact), and (**E**) the NebNext Direct Cancer Hotspot Panel (NEB). (**F**) Shown are the detection rates of mutations in the Seraseq ctDNA Complete reference Material for the AVENIO Targeted kit. The numbers in brackets reflect the number of mutations covered by the panel.

Next, we assessed the performance of five commercially available mutation assays, i.e., the AVENIO Targeted Kit (Roche, herein referred to AVENIO Targeted; sequenced on an Illumina NextSeq), the QIAseq Human Actionable Solid Tumor (QIAGEN, herein referred to QIAseq; sequenced on an Illumina NextSeq), the NebNext Direct Cancer Hotspot Panel (New England Biolabs, herein referred to NEB; sequenced on an Illumina NextSeq), the GeneRead QIAact Lung UMI Panel (QIAGEN, herein referred to QIAact; sequenced on a QIAGEN GeneReader) and the Oncomine Lung cfDNA Assay (Thermo Fisher, herein referred to Oncomine; sequenced on the Thermo Fisher IonTorrent) at a single center (MUG) (Figure 1B left leg, Table S2). To assess the sensitivity, we used for all assays 15ng of the Seraseq ctDNA reference material v2, which is a full-process plasma-like material supporting the assessment of the entire workflow from extraction through the analysis. It includes 40 clinically relevant mutations across 28 genes at VAFs, i.e., 2%, 1%, 0.5%. 0.25%, 0.125%, and a wild-type (WT) sample. Of those mutations, a range of 12-37 mutations was covered by the various assays (Table S3). As the sequencing coverage dictates the analytical sensitivity and the various run designs of the different assays yielded variable sequencing depths (median depth of 2099×, 2900×, 5075×, 8364× and 798× across targeted regions for NEB, QIAseq, AVENIO Targeted, Oncomine and QIAact, respectively), it was not surprising that the detection rates varied between the assays (Figure 3, Table S4). The Oncomine assay enabled a detection of all 13 variants covered by the panel down to a VAF of 0.25% and missed two variants at a VAF of 0.125% (Figure 3A, Table S4). AVENIO Targeted detected all variants (*n* = 15) at VAFs of 2% and 1%. At VAFs of 0.5%, 0.25% and 0.125%, still 87%, 73% and 60% of expected variants were called by the AVENIO analysis pipeline (Figure 3B, Table S4). The two fusions included in the reference material (*NCOA4-RET* and *TRP-ALK*) were detected down to a VAF of 0.5%. Detection rates for the QIAseq assay were slightly lower with 100% at VAF2%, 92% at VAF1%, 64% at VAF0.5%, 28% at VAF 0.25% and 20% at VAF0.125% (Figure 3C, Table S4). In contrast, detection rates of the QIAact assay (100% at VAF2%, 50% at VAF1%, 35.7% at VAF0.5%, 7.1% at VAF 0.25% and 0% at VAF0.125%) and the NEB assay (49% at VAF2%, 35% at VAF1%, 22% at VAF0.5%, 5% at VAF 0.25% and 5% at VAF0.125%) were considerably lower (Figure 3D,E, Table S4). These observations were not unexpected, since for example, the sequencing depth of the QIAact libraries was 10-fold lower compared to Oncomine, and for the NEB kit there was no optimized analysis pipeline available. In general, the concordance of expected and observed VAFs was high (Oncomine *pc* = 0.948, for QIAact *pc* = 0.782, QIAseq *pc* = 0.873, and AVENIO Targeted *pc* = 0.840) (Figure S3) with the exception for the NEB kit (*pc* = 0.270).

For AVENIO Targeted, we additionally tested the Seraseq ctDNA Complete reference material, which covers a slightly different set of mutations (Table S5) and comes with other VAFs (5%, 2.5%, 1%, 0.5%, and 0.1%). Using this set of reference material, the detection rate was similar to Seraseq ctDNA reference material v2, but the concordance improved (*pc = 0.945*) (Figure S3, Figure 3F, Table S4). 

#### 2.2.2. Assessment of Variant Calling Accuracy

To assess the accuracy of a test, the number of false positive (FP) mutation calls and the likelihood that a variant call is a true positive (TP) need to be considered. Here, we evaluated the background of the Seraseq ctDNA Reference Materials to calculate variant calling accuracy of the three best performing assays, i.e., AVENIO Targeted, QIAseq and Oncomine (Figure 1B right leg). With the exception of the Oncomine assay, in which two *TP53* mutations—each with one mutated read—were detected, the WT samples had none of the hotspot mutations called, indicating a high specificity for these regions. To assess the variant calling accuracy, we set 0.5%, 0.2%, and 0.1% thresholds for the limit of detection (LOD), VAFs that are commonly observed in clinical samples and should be detectable based on the vendors’ specifications. We considered SNVs and indels with VAFs higher than the LOD but not reported by SeraCare as false positives (FP). Variants included in the reference material and covered by the respective panel were only considered as true positives (TP) if the VAF was equal or greater than the LOD. For the Oncomine assay, we conducted two analyses. First, we used all variants across the panel that were called by the cfDNA Torrent Suite variant caller, i.e., a de novo calling approach. Second, we confined the analysis to a pre-filtered set of variants located in only 124 hotspots reported by the Ion Reporter Software.

**Figure 4:**
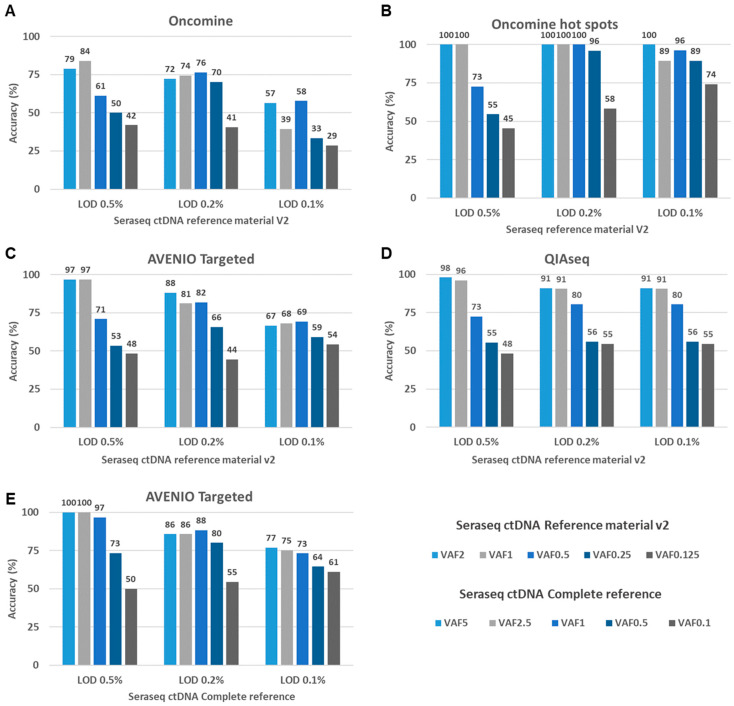
Accuracy rates of three NGS assays assessed with the Seraseq ctDNA Reference Material. (**A**–**D**) To assess the variant calling accuracy of the tested assays, we set 0.5%, 0.2%, and 0.1% thresholds for the limit of detection (LOD). Variants known to be present in the Seraseq ctDNA Reference Material v2 and called with a VAFs above the LOD were considered as true positives. Variants with VAFs higher than the LOD but not reported by SeraCare were considered as false positives. Shown are accuracy rates results for five different reference materials with various VAFs for (**A**) the Oncomine Lung cfDNA Assay (Oncomine) (**B**) a pre-filtered set of variants located in hotspot regions from the Oncomine Lung cfDNA Assay (Oncomine) (**C**) the AVENIO Targeted Kit (AVENIO), (**D**) the QIAseq Actionable Panel (QIAseq) (**E**) Accuracy of the AVENIO Targeted kit assessed with the Seraseq ctDNA Complete reference material.

At an LOD of 0.5%, variant calling accuracies ranged from 79–100% for the detection of variants with VAFs 2% and 1%, and 61–73% for variants at a VAF of 0.5%. For the detection of variants with VAFs <0.5%, accuracy decreased substantially for all assays. When lowering the LOD to 0.2% and 0.1%, respectively, a high sensitivity remained (Figure 4A–D, Table S6). However, the accuracy further decreased, in particular for the detection of variants with VAF < 0.5%, which can be attributed to an increasing number of putative FP variant calls at an LOD of 0.2 or 0.1% for all assays (Table 1 and Table S6). As for the Oncomine assay, the de novo calling approach considering all regions of the panel resulted in a higher number of FPs, leading to a decreased accuracy compared to the pre-filtered list with variants located in the hotspot regions (Figure 4A,B).

FP calls arise for a variety of technical and biological reasons, as well as stochastically. Moreover, during the synthesis of the reference material, DNA can be damaged, which can additionally contribute to noise. The Seraseq ctDNA complete reference material is generated by a gentler synthesis process (information from vendor) [26] and is therefore supposed to have a lower background than the Seraseq ctDNA reference v2. Indeed, the numbers of FPs were lower for the AVENIO Targeted kit in combination with the Seraseq ctDNA complete reference material, resulting in an improved accuracy (Table 1, Figure 4C,E).

Detailed values for sensitivity, specificity, precision and accuracy for all kits are shown in Table S6. In general, most of the FP calls ranged around a VAF of 0.2% (Figure S4, Table 1) and some of them were recurrently observed in several samples (Figure S5). The majority of FP calls were C > T transitions for AVENIO and QIAseq, in contrast to C > A transversions for Oncomine (Figure S6).

### 2.3. The Use of Diagnostic LeukApheresis (DLA) Plasma for Inter-Laboratory and -Assay Comparisons

#### 2.3.1. Comparison of ctDNA from Plasma Derived from DLA and Streck Blood Collection Tubes (BCT)

**Figure 5:**
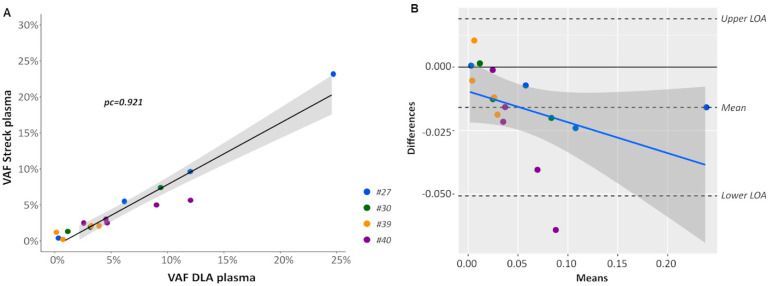
Comparison of variant call from DLA and Streck plasma. (**A**) Shown is a linear regression of variant allele frequencies (VAF) of mutations identified in plasma collected from Streck BCT and DLA including the 95% CI (gray). pc, Lin’s concordance correlation coefficient (**B**) Bland–Altman plot of differences between VAFs obtained from Streck BCT and DLA, respectively, versus the mean of the two measurements with upper and lower limits of agreement (upper/lower LOA, 95%). VAFs obtained from Streck tubes were slightly lower compared to DLA resulting in a bias of −0.0159. VAFs from different patients are plotted using a different colored symbol.

Although highly characterized synthetically generated reference material and cell lines can be used to assess and validate assay performances, the testing of patient-derived samples is preferable. However, a major challenge is the lack of sufficient amounts of patient-derived plasma to comprehensively validate and monitor assay performance of different ctDNA detection assays or at different testing sites. Recently, DLA to screen liters of blood for the presence of circulating tumor cells (CTCs) has been introduced [27,28]. During this process, large volumes of plasma (up to 100 mL) can be harvested in citrate solution (ACD-A Anticoagulant Citrate Dextrose Solution, Solution A) and potentially used for ctDNA analyses. To check the concordance of DLA plasma and plasma extracted from a conventional venous blood draw into a Streck Blood Collection Tube (BCT), we analyzed corresponding DLA and Streck BCT samples taken at the same time point from four patients with non-small cell lung cancer (NSCLC) (#27, #30, #39, and #40) using the AVENIO Expanded kit (Figure 1C). On average, 14.5 ng/mL DLA plasma (range 2.7–47.1) and 22.6 ng/mL Streck plasma (range 10.3–51.5) could be obtained. Analysis of the size distribution of cfDNA from DLA plasma revealed the same picture as from Streck plasma with the majority of fragment ranging around 160 bp and multiples thereof (Figure S7). VAFs were highly concordant (*pc* = 0.921) (Figure 5A) and a Bland–Altman plot was created for further analyzing the agreement of VAFs from Streck BCT and DLA. Even if there was a bias (−0.0159) towards lower VAFs in Streck BCT, all but one of the values lay within the 95% limits of agreement (Figure 5B).

#### 2.3.2. Inter-Laboratory and -Assay Comparison Using DLA Plasma

**Figure 6:**
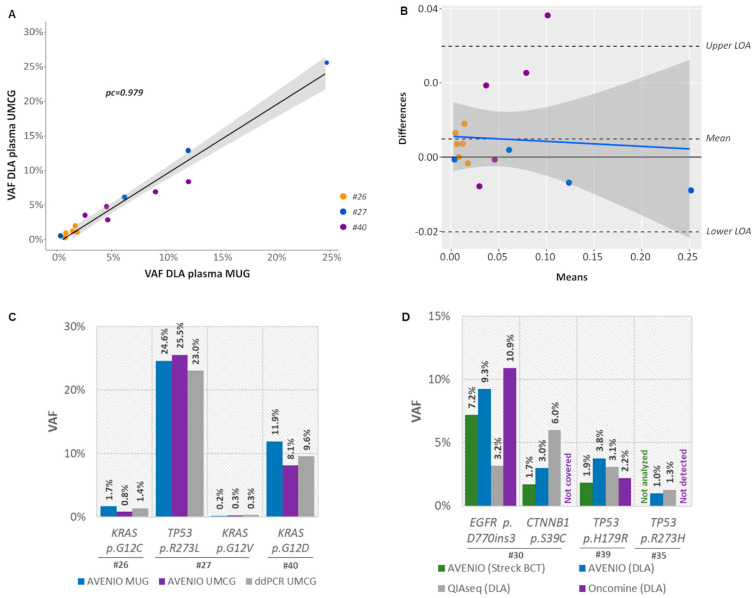
The use of DLA plasma for inter-laboratory and -assay comparison. (**A**) Shown is a linear regression of variant allele frequencies (VAF) of mutations identified in plasma collected from Streck BCT and corresponding DLA samples using the AVENIO Expanded Kit including the 95% CI (gray). pc, Lin’s concordance correlation coefficient. (**B**) Bland–Altman plot of differences between VAFs obtained at MUG and UMCG versus the mean of the two measurements with upper and lower limits of agreement (upper/lower LOA, 95%). There was only one outlier outside the LOA resulting in a bias of 0.0049. VAFs from different patients are plotted using a different colored symbol. (**C**) VAFs of *KRAS* codon 12 and *TP53* codon 273 mutations validated by ddPCR (**D**) VAFs of variants detected in DLA and Streck BCT plasma using AVENIO Expanded Kit, the Oncomine Lung cfDNA Assay and the QIAseq Human Actionable Solid Tumor Assay.

For an inter-laboratory comparison of the AVENIO Expanded kit, three DLA samples (#26, #27, and #40) were additionally analyzed at UMCG. Except for a single mutation in patient #26 (*BRCA2*: c.7781A>G, p.Lys2594Arg, VAF% 0.62% detected at MUG), all variants identified at MUG were also identified at UMCG (93% concordance) with highly concordant VAFs (*pc* = 0.979) (Figure 6A, Table S7). A Bland–Altman plot revealed a high agreement with only one outlier outside the upper limit of agreement (bias 0.0049) (Figure 6B). Hotspot mutations in *KRAS* (codon 12) and *TP53* (codon 273) were validated with ddPCR and confirmed the presence and VAFs of these variants (Figure 6C).

To check the concordance of different ctDNA NGS platforms, we analyzed three DLA samples (#30, #39 and #35) with the Oncomine Lung cfDNA Assay and the Human Actionable Solid Tumor Panel in addition to the AVENIO Expanded kit. A total of four mutations were covered by all three assays. Of these, three variants were consistently detected across all platforms with similar VAFs, whereas a low-level *TP53* mutation was identified by AVENIO Expanded (VAF0 0.99%) and QIAseq (VAF1.3%), but its presence was not confirmed by Oncomine (Figure 6D, Table S7).

## 3. Discussion

With the growing number of predictive molecular biomarkers in oncology, NGS technologies increasingly complemented conventional techniques such as immunohistochemistry (IHC), fluorescence in situ hybridization (FISH), and RNA-based gene fusion detection, which are routinely applied to FFPE tissue biopsies [29]. Moreover, the use of cfDNA is a promising alternative to tissue biopsies and a variety of cfDNA-based NGS assays are now commercially available and are being increasingly adopted in clinical practice [14,16]. Yet, the validity of these products as well as the clinical utility of ctDNA in the management of patients with cancer has yet to be proven [30]. Within the framework of Cancer-ID, which comprises a consortium of experts, companies and institutions for blood-based biomarker validation, assay development, clinical sciences and bioinformatics, we conducted a comprehensive evaluation of reference material and various mutation analysis platforms for liquid biopsy profiling. In three experimental setups, we tested the use of commercially available reference materials specifically designed for ctDNA testing and plasma derived from DLA and corresponding venipuncture blood drawn for inter- and intra-assay as well as intra- and inter-laboratory comparisons.

Our data demonstrate that reference materials with predetermined VAFs are extremely useful for the performance assessment of both PCR- and NGS-based mutation analysis platforms. The Seraseq reference materials used in this study cover selected clinically relevant mutations, many of which currently guide treatment decisions. We first used the Seraseq ctDNA complete reference material to measure reproducibility between and within runs for ddPCR, the MassARRAY and three NGS assays. Despite the use of only three replicates, we obtained meaningful results. At a VAF of 1%, the CV ranged from 9–31%, with the MassARRAY being the assay with the lowest variability. However, we showed that such comparisons critically depend on the amount of template DNA and demonstrated that a reduced input DNA compromised assay precision. Using input amounts of 8ng and higher, the allele frequency estimates were sufficiently accurate, but variability increased significantly when using only 2 or 4 ng for ddPCR. The observed variability could to a large extent be attributed to the expected random error of VAF calling indicating that all tested assay yielded robust results. For MassARRAY the observed CV was lower than expected, which can be attributed to the fact that the target signal-to-noise ratio (SNR) is normalized to six internal controls. Considering the variance of the target SNR without normalization, the CV would be in the expected range. The total DNA input amount is also a crucial determinant of sensitivity, as the total number of molecules assayed determines the ability to detect mutant copies. We then demonstrated the importance of the sequencing depth for NGS assays. For example, to detect three mutated copies at an 0.1% LOD, a minimum of 20 ng of input cfDNA is required in combination with a 3000x sequencing depth. Although lower amounts of cfDNA can be used, the %LOD needs to be adjusted depending on the input amount. It is of note that, when using UMIs for error correction, increasing DNA input requires a proportional increase in sequencing to obtain the same number of average replicates per UMI. Nonetheless, preanalytical handling and high efficiency for cfDNA isolation is critical for the detection of low-frequency ctDNA mutations [31,32]. Finally, for NGS approaches, a well-established bioinformatics analysis workflow can make a difference for the assay performance. This is highlighted by our platform comparison in study setup B. The two best performing assays of this evaluation, the Oncomine and the AVENIO platform, which enabled mutation detection down to 0.125%, both provide an end-to-end solution and come with a generic analysis workflow to provide support for the detection of genetic alterations. Although this also applied to QIAact, the sequencing depth for this assay was significantly lower, which is a likely cause for the lower performance. For the QIAseq assay, we used the specifically developed open source smCounter analysis pipeline, which also enabled satisfying detection rates. For the NEB assay, there was no optimized pipeline available at the time of testing, thus requiring personnel with bioinformatics skills to convert raw sequencing data into annotated variants. Using the MuTect2 algorithm, which was actually developed for tumor/normal pairs, many mutations were not called, although some of the variants were clearly identified after visual evaluation of the BAM file using the IGV browser (Table S4). Therefore, an optimized analysis pipeline might improve the recall rate of this assay.

To demonstrate the performance of NGS liquid biopsy tests, determination of the analytical specificity in addition to sensitivity is crucial. With respect to accuracy of the variant calling, we observed a high specificity for mutations located in the hotspots covered in the SeraSeq reference material. Except for the Oncomine assay with two FP reads, the AVENIO and the QIAseq assay did not show any FP reads in the WT reference samples at positions with known variants. Yet, in all kits, putative FP variant calls were observed outside these regions. Whether these FP calls originate from technical or biological sources cannot be determined, even though the most recent version of the Seraseq reference material led to a lower number of FPs. Our evaluation demonstrated that variants with allele frequencies of 1% and higher can be detected with a very high accuracy at an LOD of 0.5%. Yet, as expected, an overall reduction in accuracy was observed for calling low allele frequency mutations or at lower LODs. Although not investigated here, the use lower input amounts might further affect accuracy. Similar data were reported from Stetson and colleagues, who tested four centralized NGS assay vendors using replicates of plasma samples and matched tumor–normal tissue pairs [25]. Their analyses revealed substantial variability among the ctDNA assays and, as with our observation, the majority of the discordance was observed below a 1% VAF. Taken together, these data indicate that despite the use of UMIs, the biggest technical challenge of cfDNA assays is still the ability to accurately discriminate between low allele frequencies of true somatic variants and technical artifacts. Accuracy is likely to be further improved by bioinformatics filters, but currently, a maximization of sensitivity can only be achieved at the expense of specificity. Therefore, low frequency variants should be interpreted with caution and de novo variant calling might require a more stringent LOD in contrast to hotspot mutations or a tumor-informed approach, which can achieve a higher sensitivity and specificity. Nonetheless, the accurate screening for clinically relevant somatic mutations is a crucial step in precise clinical diagnosis and targeted therapy. Therefore, reports which guide treatment decision making should contain specific information on the quality metrics of the assay and the maximum achievable LOD with respect to assay performance, sequencing depth, and input amount [29,32,33].

In addition to the in-house performance assessment of a test, laboratories delivering ctDNA tests need to conduct validation processes to ensures that a test is being performed correctly in the course of external quality assessment (EQA) [34,35], i.e., the assessment and monitoring of the performance of individual laboratories for specific tests or measurements. Although there is a very good acceptance for patient-like reference materials, testing of patient-derived samples is preferable for EQA and proficiency testing. Yet, patient samples are limited, often poorly characterized and lack breadth in terms of the mutations evaluated. One option for obtaining access to large volumes of plasma is DLA [28,36]. We demonstrated a high concordance of mutations and respective VAFs identified from DLA plasma and plasma from conventional blood draws. Furthermore, we used DLA plasma for inter-assay comparisons of the AVENIO, Oncomine and QIAseq platforms and obtained highly consistent results. Likewise, an inter-laboratory comparison of the AVENIO Expanded Kit using DLA plasma performed at MUG and UMCG revealed a high concordance. Taken together, these data indicate that DLA plasma is an attractive option for overcoming the lack of patient material for EQA.

In summary, our data demonstrate that after comprehensive evaluation and validation using reference material, commercially available mutation analysis platforms can be used for liquid profiling and most of them enable the detection of low-frequency variants, whereas end-to-end solutions, in particular AVENIO and Oncomine, currently provide better performances.

## 4. Materials and Methods 

### 4.1. Reference Materials and Patient Samples

Two sets of reference materials were used. The Seraseq^®^ ctDNA Reference Material v2 (SeraCare Life Sciences, Milford, MA, USA) harbors 40 variants in 28 genes at specified VAFs (2%, 1%, 0.5%, 0.25%, 0,125%, 0%) targeting cancer-relevant somatic mutations, whereas GM24385 human genomic DNA serves as background wild-type material (Table S3). Likewise, the Seraseq^®^ ctDNA Complete™ (SeraCare Life Sciences) reference material covers 25 clinically relevant variants (12 SNVs, 5 deletions, 2 insertions, 3 CNAs, 3 fusions) across 16 genes (Table S5). Commonly, these are prepared by blending synthetic DNA encoding individual mutations with GM24385 genomic DNA at specified VAFs (5%, 2.5%, 1%, 0.5%, 0.1%, 0%). The mixture is sized to mimic cfDNA fragment size profiles. Digital droplet PCR (Bio-Rad, Hercules, CA, USA) was used to confirm the VAF for each variant. The Seraseq ctDNA reference materials were used as purified ctDNA (study setup A) or encapsulated in a plasma-like matrix format (study setup B). Moreover, plasma derived from Cell-Free DNA BCT (Streck, La Vista, NE, USA) and/or DLA from six NSCLC patients were obtained from the University Medical Center Groningen. Blood for DLA was anticoagulated with citrate dextrose solution A (ACD-A solution; Fresenius HemoCare) and processed as reported previously [28,36].

### 4.2. DNA Isolation 

For study setup B, DNA was isolated from 5 mL Seraseq ctDNA reference material v2 with the QIAamp Circulating Nucleic Acid Kit (QIAGEN, Hilden, Germany) and the concentration was measured by Qubit dsDNA High Sensitivity Assay (ThermoFisher, Waltham, MA, USA). For study setup C (Figure 1), cfDNA from Streck BCT and DLA plasma samples was extracted from 1ml using the same extraction method. Size distribution was assessed on a High Sensitivity DNA Chip on a Bioanalyzer (Agilent, Santa Clara, CA, USA).

### 4.3. Mutation Analysis Platforms

For the various study setups, several commercially available mutation analysis assays were used. All assays were performed in accordance with the manufacturer’s recommendation and a detailed description can be found in the Supplementary Materials section. The centers involved in the study and the respective assay performed at each center are listed in Table 2.

### 4.4. Statistical Analysis

Data analysis and visualization were performed using Excel (Microsoft Corporation, Redmond, WA, USA), Prism (version 7.0, Graphpad Software, Inc., San Diego, CA, USA) and R Studio software (version 3.5.2, RStudio, Inc., Boston, MA, USA). Intra-run precision was evaluated by testing the VAF1% sample in triplicate in a single complete mutational analysis workflow. Inter-run reproducibility was assessed by testing the sample in three separate mutational analysis workflows. *Coefficient of variation* is defined as the ratio of the standard deviation to the mean. Shot (Poisson) noise was calculated by sqrt((1 − expected VAF)/(DNA copies * expected VAF)). The confidence interval variant calling precision in relation to the input amount was calculated using the *t* = BINOM.INV() function tin Excel. To assess the concordance of expected and observed VAFs, we calculated the *Lin´s concordance correlation coefficient*, which assesses the degree of agreement between two continuous variables using the *epiR* package package (function *epi.ccc*) in R Studio (version 1.2.1335). Plots for false positive variant calls were generated with the R package maftools version 2.0.16.

## 5. Conclusions

The development of a custom liquid profiling test requires significant operational and bioinformatics infrastructure. Thus, labs may use vendor solutions, which typically target selected clinically relevant and well-characterized hotspot regions. However, the choice of an optimal panel is challenging and depends on many factors, including the content, tumor type, results required, technical efficiency, size and of course cost. Our data demonstrate that a range of commercially available mutation analysis kits can be used for liquid profiling but vary with respect to performance and accuracy. Therefore, extensive validation of the test performance and a determination of the LOD is required before offering these tests in clinical routine practice. To this end, patient-like reference material is extremely useful. However, in addition to in-house validation, a verification of test results in the framework of EQA, preferentially with patient material, e.g., DLA plasma, is crucial. Nevertheless, in addition to clinical utility, we still lack evidence-based guidelines regarding who should order a test and when. Moreover, the relevance of low-frequency variants in clinical practice remains debated. Based on prior clinical validation studies, large prospective interventional trials are currently being conducted to provide evidence of the clinical utility of liquid profiling from cfDNA.

## Figures and Tables

**Table 1:** False positive variant calls at various limits of detection (LOD).

LOD	0.50% *	0.20% *	0.10% *	Average VAF (Range) at LOD 0.1%
AVENIO Targeted Seraseq ctDNA reference material v2	0.8	5.6	13.6	0.21% (0.1–0.7)
AVENIO Targeted Seraseq ctDNA Complete	0	4.4	10.2	0.21% (0.1–0.4)
Oncomine (all regions)	5.2	9.8	35	0.23% (0.1–1.5)
Oncomine (hot spot regions)	0	0.2	1.8	NA
QIAseq	2.4	4.2	4.2	0.73% (0.2–2.4)

* Average numbers of false positive variant calls for all tested reference materials.

**Table 2:** Participating Cancer-ID sites and performed mutation analysis assays.

Site	ID	Assay	Sequencing/ Assay Platform	Study Set up
Agena	Agena	UltraSeek lung panel (Agena BioScience)	MassARRAY	A
BAYER	BAYER	Digital droplet PCR	Bio-Rad	A
CRUK Manchester Institute	UNIMAN	SureSelectXT Custom Kit (Agilent)	Illumina	A
Institute of Human Genetics, Medical University of Graz	MUG	AVENIO ctDNA Targeted Kit (Roche)	Illumina	A, B
		AVENIO ctDNA Expanded Kit (Roche)		C
		QIAseq Human Actionable Solid Tumor Assay (QIAGEN)		B, C
Institute of Pathology, Medical University of Graz	MUG	QIAact Lung UMI Panel (QIAGEN)	GeneReader	A, B
		Oncomine cfDNA Lung Panel (Thermo Fisher)	IonTorrent	B, C
Department of Pathology, University Medical Center Groningen	UMCG	Digital droplet PCR	Bio-Rad	A, C
		AVENIO ctDNA Expanded Kit (Roche)	Illumina	C

## Supplementary Materials

The following are available online at https://www.mdpi.com/2072-6694/12/6/1588/s1, Figure S1. Intra-run precision and inter-run reproducibility of three NGS- and three qPCR-based mutation analysis assays. Figure S2. Association of input mass quantities and variability in mutation detection of ddPCR. Figure S3. Concordance of expected and observed variant allele frequencies (VAF). Figure S4. Boxplots showing the distribution of variant allele frequencies (VAF) of variant calls that are not reported in the reference material. Figure S5. Positions with recurrent false positive variant calls. Figure S6. Summary of false positive (FP) variant calls. Figure S7. Size distribution of cfDNA extracted from Streck and DLA plasma. Table S1. Inter-run and intra-run variability of three NGS- and three qPCR-based mutation analysis assays. Table S2. Content and amount of input material of NGS assays assessed in the study setup B. Table S3. Mutations covered by each NGS panel in Seraseq ctDNA Reference Material v2. Table S4. Detection rates and QC metrics of NGS panels tested with Seraseq ctDNA reference material v2. Table S5. Mutations covered by the AVENIO Targeted Kit in Seraseq ctDNA Complete reference material. Table S6. Accuracy, precision, sensitivity and specificity of three NGS panels tested with Seraseq ctDNA v2 reference material. Table S7. Inter-laboratory and inter-assay comparison using plasma derived from DLA and Streck BCT.
