# Supplementary material for: Technical Evaluation of Commercial Mutation Analysis Platforms and Reference Materials for Liquid Biopsy Profiling
Source: Cancers (Basel). 2020 Jun 16;12(6):1588. doi: 10.3390/cancers12061588 (PMC7352370; doi:10.3390/cancers12061588)
Supplement: Supplementary file 1 [file cancers-12-01588-s001.zip › cancers-773680-supplementary-final.docx]

**Supplementary Materials:**

Technical Evaluation of Commercial Mutation Analysis Platforms and Reference Materials for Liquid Biopsy Profiling

Sabrina Weber, Benjamin Spiegl, Samantha O. Perakis, Christine M. Ulz, Peter M. Abuja, Karl Kashofer, Paul van der Leest, Maria Aguirre Azpurua, Menno Tamminga, Dan Brudzewsky, Dominic G. Rothwell, Sumitra Mohan, Alexander Sartori, Rita Lampignano, Yves Konigshofer, Markus Sprenger-Haussels, Harriet Wikman, Inger R. Bergheim, Vera Kloten, Ed Schuuring, Michael R. Speicher and Ellen Heitzer

SureSelect XT Custom kit (Agilent)

The SureSelect Custom Kit is a custom-designed small cell lung cancer panel and covers exon regions of 110 genes (Table SX; 667 kb). This panel uses hybrid capture-targeted enrichment techniques. Whole genome libraries were generated from 20 ng Seraseq ctDNA Complete DNA. In brief, end repair and A-tailing was performed using the NEBNext Ultra II End Repair/dA-Tailing Module (New England Biolabs), adapter ligation and library amplification was carried out with KAPA HiFi HotStart PCR Kits (Kapa Biosystems) with indexing using NEBNext Index Primers for Illumina (New England Biolabs). The libraries were assessed using a Nanodrop as well as an agarose gel. Further, 1μg of whole genome indexed library was taken forward as input for custom capture (110-gene panel) on SureSelectXT Reagent Kits (Agilent) according to the manufacturer’s instructions. Captured libraries were amplified using KAPA HiFi HotStart PCR Kits (Roche) and quantified using the KAPA library quantification qPCR kit (Roche). Libraries were paired end sequenced on an Illumina NextSeq 500, 2 × 150 bp High Output V2 kit (Illumina).

On average, 101.67 M raw read pairs were obtained per sample (range 55.56 M–186.06 M) with mean unique depth of 2890.9 reads (range 2455x–3237x) after duplicate removal for the Seraseq ctDNA Complete samples. FASTQ files were generated from the sequencer’s output using Illumina bcl2fastq2 software (v.2.17.1.14, Illumina) with the default chastity filter to select sequence reads for subsequent analysis. All sequencing reads were aligned to the human genome reference sequence (GRCh37) using the BWA (v.0.7.12) MEM algorithm. Picard tools (v.2.1.0) were used to mark/remove PCR duplicates and to calculate sequencing metrics. Variants were called using two callers- MuTect2 (GATK) and a commercial software Biomedical workbench v5.0 (Qiagen) and variants calls were kept only if called by both variant callers.

| **SureSelect Custom Kit Gene List** | | | | |
| --- | --- | --- | --- | --- |
| ***ABL1*** | ***EGFR*** | ***GATA3*** | ***MYCL*** | *RAD51* |
| *ABL2* | *EP300* | *GNAS* | *NF1* | *RAD51C* |
| *AKT1* | *EPHA3* | *GRIN2A* | *NFATC2* | *RASSF1* |
| *AKT2* | *EPHA5* | *HGF* | *NFE2L2* | *RB1* |
| *ALK* | *ERBB2* | *HRAS* | *NOTCH1* | *RBL1* |
| *APC* | *ERG* | *IDH1* | *NOTCH2* | *RBL2* |
| *AR* | *EZH2* | *IDH2* | *NOTCH3* | *RET* |
| *ATM* | *FANCA* | *JAK2* | *NRAS* | *ROS1* |
| *ATR* | *FANCC* | *KDM6A* | *NTRK1* | *RUNX1T1* |
| *AURKA* | *FANCD2* | *KEAP1* | *NTRK3* | *SETBP1* |
| *BAP1* | *FANCE* | *KIT* | *PALB2* | *SF3B1* |
| *BARD1* | *FANCF* | *KMT2A* | *PARP1* | *SLIT2* |
| *BAX* | *FANCG* | *KMT2D* | *PARP2* | *SRC* |
| *BRAF* | *FANCL* | *KRAS* | *PARP3* | *STK11* |
| *BRCA1* | *FBXW7* | *LIFR* | *PARP4* | *TMPRSS2* |
| *BRCA2* | *FGFR1* | *MET* | *PDE4DIP* | *TP53* |
| *BRIP1* | *FGFR2* | *MSH2* | *PDGFRA* | *TP73* |
| *CDK4* | *FGFR3* | *MSH6* | *PHF6* | *TSC1* |
| *CHEK2* | *FHIT* | *MTOR* | *PIK3CA* | *TSC2* |
| *COL22A1* | *FLT1* | *MUC16* | *PRKDC* | *VEGFA* |
| *CREBBP* | *FLT3* | *MUTYH* | *PTEN* | *WT1* |
| *CTNNB1* | *FLT4* | *MYC* | *RAD50* | *ZNF521* |

AVENIO ctDNA Targeted Kit (Roche)

AVENIO ctDNA Targeted is used for pan-cancer research applications and contains 17 genes across 81kb, including genes for NSCLC, CRC and other cancer types aligned with the U.S. National Comprehensive Cancer Network (NCCN) Guidelines. This panel used hybrid capture-targeted enrichment techniques. The panel includes SNVs in *ALK, APC, BRAF,BRCA1,BRCA2,DPYD, EGFR, ERBB2, KIT, KRAS, MET, NRAS, PDGFRA, RET, ROS1, TP53* and *UGT1A1*; Indels in predefined regions of *ALK, APC, BRAF, EGFR,ERBB2, KIT, MET*; Fusions in *ALK, RET* and *ROS1*, additionally CNVs *EGFR*, *ERBB2* and *MET*. The workflow involved integrated digital error suppression (iDES) strategies by combining molecular barcodes with in silico error suppression techniques (1, 2). According to the vendor, the assay detects SNVs with VAF of 0.5% with >99% sensitivity and specificity using 10-50ng of mixed cell lines and a minimum of 40M reads. VAFs with 0.1% can also be detected.

Libraries were prepared from 15ng (study setup B) and 20ng (study setup A) DNA of SeraCare reference material v2 and Seraseq® ctDNA Complete, respectively, according to AVENIO ctDNA Analysis Reagent Workflow User Guide (version 1.0.0). Assessment of enriched library quality was done with Bioanalyzer High Sensitivity kit (Agilent). According to Roche´s recommendations a total of 16 libraries were pooled according to measurements with Invitrogen Qubit dsDNA HS Assay Concentrations (Thermo Fisher). The pools were quantified with qPCR and sequenced on an Illumina NextSeq 550 platform with a High Output V2 300 cycles Kit in paired end mode. To increase sequencing complexity, 10% PhiX Sequencing Control V3 were spiked to the pool. On average, 22.2M raw read pairs were obtained per sample (range 20.2M- 27.5M) with mean unique depth of 5075 reads (range 4544x-6288x) after consensus read generation for the SeraCare reference material v2 (study setup B). For the Seraseq® ctDNA Complete samples an average of 20.0M raw read pairs per sample (range 18.2-23.5M) with a mean depth of 4002 reads (range 2967x-4603x) were obtained (study setup A). Sequencing data was analyzed using the AVENIO ctDNA Analysis Software (Roche, version 1.1.0), variant calls were generated with a customized workflow using defined somatic variant filter settings. The filter removed variants present with >1% mutated population allele frequency listed in frequency databases (ExAC, gnomAD, 1000genomes) along with common SNPs as defined in dbSNP.

The AVENIO ctDNA Expanded Kit (Roche)

The AVENIO ctDNA Expanded Kit is a liquid biopsy assay with a 77 gene panel containing genes in U.S. National Comprehensive Cancer Network (NCCN) Guidelines1 and emerging cancer biomarkers.

Like the Targeted kit The workflow involved integrated digital error suppression (iDES) strategies by combining molecular barcodes with in silico error suppression techniques (1, 2). According to the vendor, the assay detects SNVs with VAF of 0.5% with >99% sensitivity and specificity using 10-50ng of mixed cell lines and a minimum of 60M reads. VAFs with 0.1% can also be detected.

For study set-up C an average of 29.0ng (range 15.9-50.6ng) and 19.9ng (range 7.8-50.2ng) was used for Streck BCT and cfDNA from DLA plasma. An average of 29.5M raw (range 25M-32.5M) and 22.2M (range 10M-31.8M) raw read pairs were obtained for patient-derived samples resulting in a median UMI coverage of 3221x (range 1224x-5988x) for Streck BCT and 2322x (range 306x-6448x) for DLA plasma. Quality control was performed as described above and libraries were sequenced in pools of 11. The same analysis setting and filter criteria as described for the Targeted Kit were used.

GeneRead^TM^ QIAact Lung UMI Panel (QIAGEN)

GeneRead QIAact Lung UMI Panel is designed to enrich specific target regions in selected genes (*AKT1, ALK, BRAF, DDR2, EGFR, ERBB2/HER2, ESR1, FGFR1, KIT, KRAS, MAP2K1, MET, NRAS, NTRK1, PDGFRA, PIK3CA, PTEN, RICTOR, ROS1*). This panel integrates UMI technology and a gene-specific, primer-based target enrichment process to enable sensitive variant detection of targeted regions by NGS on the GeneReader NGS System. The panel comes with a sample-to-insight NGS workflow including full bioinformatics analysis and interpretation. Based on the vendor’s information the assays is supposed to accurately, consistently and robustly detecting cfDNA at 1% sensitivity.

15ng of Seraseq ctDNA Reference Material v2 was used for library preparation. The library preparation was conducted according to manufacturer’s handbooks GeneRead QIAact Lung UMI Panel for library preparation, GeneRead Clonal Amp Q Kit for clonal amplification and GeneRead UMI Advanced Sequencing Q Kit for sequencing. Quality control was performed using the QIAxcel DNA High Resolution Kit. An average of 3.6M (range 3.2M-4.2M) sequencing raw reads were obtained after sequencing on the GeneReader platform. Raw data were analyzed using QCI-Analyze (QIAGEN), which includes optimized read alignment, variant calling, filtering and integrated visualization. After consensus read generation target regions were covered with an average of 798 reads (range 664-969).

QIAseq^TM^ Human Actionable Solid Tumor Assay (QIAGEN)

The panel enriches 773 variant positions in 12 exons. QIAseq digital sequencing enables by UMIs to remove PCR duplicates. QIAGEN states > 90% sensitivity for 1% NA12878 SNP and indel on typical coding region with false positive less than 15 per mega base region when variants are detected with tiled primer design to cover complete coding region of each gene.

Libraries were prepared from 15ng SeraCare reference material v2 DNA according to the QIAseq targeted DNA Panel Handbook (QIAGEN, Version 05/2017). The DNA template was enzymatically fragmented, end-repaired, A-tailed according to the protocol for cfDNA. Target regions were enriched by 651 target-specific primers. Finally, library amplification was done in a universal PCR with 23 cycles. The library size was checked using Bioanalyzer High Sensitivity DNA Kit (Agilent Technologies). For quantification purposes, the QIAseq Library Quant Assay (QIAGEN) was used according to manufacturer’s instructions. A total of 6 libraries were pooled equimolarly and sequenced on a Mid Output Kit on the Illumina NextSeq 550 platform in a paired end mode (2x150 bp). On average, 22.4 million raw reads were obtained per sample (range 18.M- 28.6M) and a mean depth of 2900 consensused reads (range 1899-3412). For study set-up C an average of 29.04ng (range 15.9-50.6ng) and 19.9ng (range 7.8-50.2ng) was used for Streck BCT and cfDNA from DLA plasma. An average of 29.5M (range 25-32.5M) and 22.2M (range 10-31.8M) raw read pairs were obtained for patient-derived samples resulting in a median UMI coverage of 3221 (range 1224-5988) for Streck BCT and 2322.4 (range 306-6448) for DLA plasma.

Sequencing data was analyzed using GeneGlobe Analysis Hub (QIAGEN), which supports the analysis of QIAseq NGS library kits and target enrichment panels. The pipeline processes the UMI information to distinguish between true variants and sequencing errors based on smCounter V1(3). All variants that did not pass the predefined quality criteria from smCounter were dismissed. All detected variants were visually checked using Integrative Genomics Viewer (IGV) (version 2.3.58)

NEBNext Direct Cancer HotSpot Panel for Illumina (New England Biolabs Inc.)

The NEBNext Direct^TM^ Cancer Hotspot Panel panel enriches 190 common cancer targets from 50 genes. NEBNext Direct employs a fast hybridization-based workflow that combines capture with library preparation. False positive variants are reduced by the use of UMIs. The vendors demonstrate a 100% detection rate of 168 truth variants present across a range of 2-100% VAF.

Library preparation was performed using 15ng SeraCare reference material v2 following the manufacturere´s recommendation (manual version 2.0). The library size was checked using Bioanalyzer High Sensitivity DNA Kit (Agilent Technologies). For quantification purposes, qPCR using Illumina adapter-specific primers were conducted. Libraries were pooled equimolarly and sequenced on the Illumina NextSeq 550 platform using a Mid Output Kit in a 75bp paired end mode. On average, 24.8M raw reads were obtained per sample (range 23.5M- 26.4M). FASTQ files were generated by the sequencing run for all UMI reads. UMI sequences were added to the BAM files using the AnnotateBamWithUmis function from the fgbio package (<https://fulcrumgenomics.github.io/fgbio/>) resulting in a mean MT depth of 2099 reads (range 1,217- 4361). Duplicate reads were marked with the MarkDuplicates function from Picard (GATK). Variant calling was performed using the GATK MuTect2 pipeline (Broad Institute, <https://software.broadinstitute.org/cancer/cga/mutect>) using the tumor-only mode and VCF files were annotated with annovar. Moreover, ​ all positions with variants included in the SeraCare reference material v2 covered by the panel were visually checked using Integrative Genomics Viewer (IGV) (version 2.3.58).

Oncomine Lung cfDNA Assay

Ion Torrent NGS libraries were prepared using the Oncomine Lung cfDNA^TM^ Assay (Thermo Fisher Scientific, CatNr: A31149) which covers >150 hotspots in 11 genes (*ALK, BRAF, EGFR, ERBB2, KRAS, MAP2K1, MET, NRAS, PIK3CA, ROS1*, and *TP53*) and implements single molecule barcoding. According to the manufacturer, the Oncomine Lung cfDNA Assay has a flexible detection limit down to 0.1% or 1 mutant copy in a background of 1,000 wild-type copies. To achieve 0.1% LOD, 20ng of input cfDNA is required. Libraries were prepared using the included multiplex PCR according to the manufacturer´s recommendation. Templates were automatically prepared and loaded on Ion 540 chips by the Ion Chef instrument and subsequently sequenced on the Ion S5XL platform using the 200bp workflow. On average, 5.8M raw reads were obtained per sample (range 4.4M- 8.5M). Sequencing data were analysed using the Torrent Variant caller (<https://github.com/iontorrent/TS>, version 5.10) software. For the assessment of variant calling accuracy, we conducted two analyses. First, we used all variants across the panel that were called by TVC, i.e. a *de novo* calling approach. Second, we confined the analysis to the Oncomine Hotspot file provided with the assay, containing the 124 hotspots that would be reported by the Ion Reporter Software.

MassARRAY (Agena Bioscience)

UltraSEEK™ Lung Panel is a liquid biopsy test for the MALDI-TOF-based MassARRAY System that detects 74 variants across 5 genes (*BRAF, EGFR, ERBB2, KRAS, PIK3CA*) and 3 variant types (SNV, insertion, deletion) that are relevant to NSCLC and compliant with the U.S. National Comprehensive Cancer Network (NCCN) Guidelines. UltraSEEK uses a single multiplex PCR, followed by mutation-specific single base extension reaction which utilizes a single mutation-specific chain terminator labeled with a moiety for solid phase capture. PCR was performed in 70 µl and enabling a maximum of 35 µl of sample. For the assessment of inter- and intrarun variability using Seraseq® ctDNA Complete 15ng were analyzed according to the UltraSEEK Panels User Guide. To this end the reference material was pre-diluted and pooled in a way that all three replicate PCRs were generated from the same pool with the highest possible volume enabling the replicates with very low variance. After the capture, cleaning, and elution process, the extension products were automatically transferred to a SpectroCHIP® Array and loaded into the MassARRAY Analyzer. Data acquired by the MassARRAY Analyzer were processed by MassARRAY Typer and UltraSEEK Report software. Briefly, a positive signal produces a peak on the MassARRAY at the expected mass of a certain height. This peak is related to the local background noise, so that the signal-to-noise ratio (SNR) rather than the signal level is used as reporter. At the same the corresponding SNR signals of 6 internal controls were determined, which monitor PCR efficiency. The target SNR was finally normalized with the SNR of the controls.

ddPCR (Bio-Rad, performed at Bayer)

For each study set-up A, ddPCR assays for *BRAF* V600E, *EGFR* T790M, *EGFR* L858R, *KRAS* G12C and *KRAS* G12D were performed using the Bio-Rad QX200™ platform included no template controls. PCR set-up was performed according to the manufacturer´s recommendation. To assess the impact of input mass on the variant calling precision, various amounts of the Seraseq® ctDNA Complete (8ng, 4ng and 2 ng) were used. Data were analyzed using the QuantaSoft™ analytical software v1.7.4 (Bio-Rad). Fluorescent signals of negative controls were recorded as background to establish cut-off levels. Results were expressed as both number of mutants/ccfDNA eluates (µL) as well as variant allele frequency (VAF; ratio between no. of mutant and wild type droplets after correction using the Poisson distribution).

ddPCR (Bio-Rad, performed at UMCG)

For mutation detection in ccfDNA extracted from plasma samples, ddPCR assays for *BRAF* V600E, *EGFR* T790M, *EGFR* L858R, *KRAS* G12C and *KRAS* G12D (study set-up A) and the *KRAS* G12/G13 screening and *TP53* R273L assay (study set-up C) were performed using the Bio-Rad QX200™ platform including positive, wild type and no template controls. PCR set-up was performed according to the manufacturer´s recommendation. DdPCR analyses were performed on 8.8µL of ccfDNA eluates extracted from 2mL plasma with variable concentration as measured by Qubit according to the manufacturer´s instructions. Data were analyzed using the QuantaSoft™ analytical software v1.7.4 (Bio-Rad). Positive, wild type and no template controls were used to establish cut-off levels. Results are expressed as VAF based on the ratio between mutant and wild type droplets after correction using the Poisson distribution (calculated by the Quantasoft software). As precautionary measure, samples were considered positive when 3 or more FAM/HEX positive droplets were detected with no positive droplets in the no-template and wild type controls. All molecular testing was performed in the ISO15189-accredited laboratory of molecular pathology at the UMCG. All standard precautions were taken to avoid contamination of amplification products using separate laboratories for pre- and post-PCR handling.


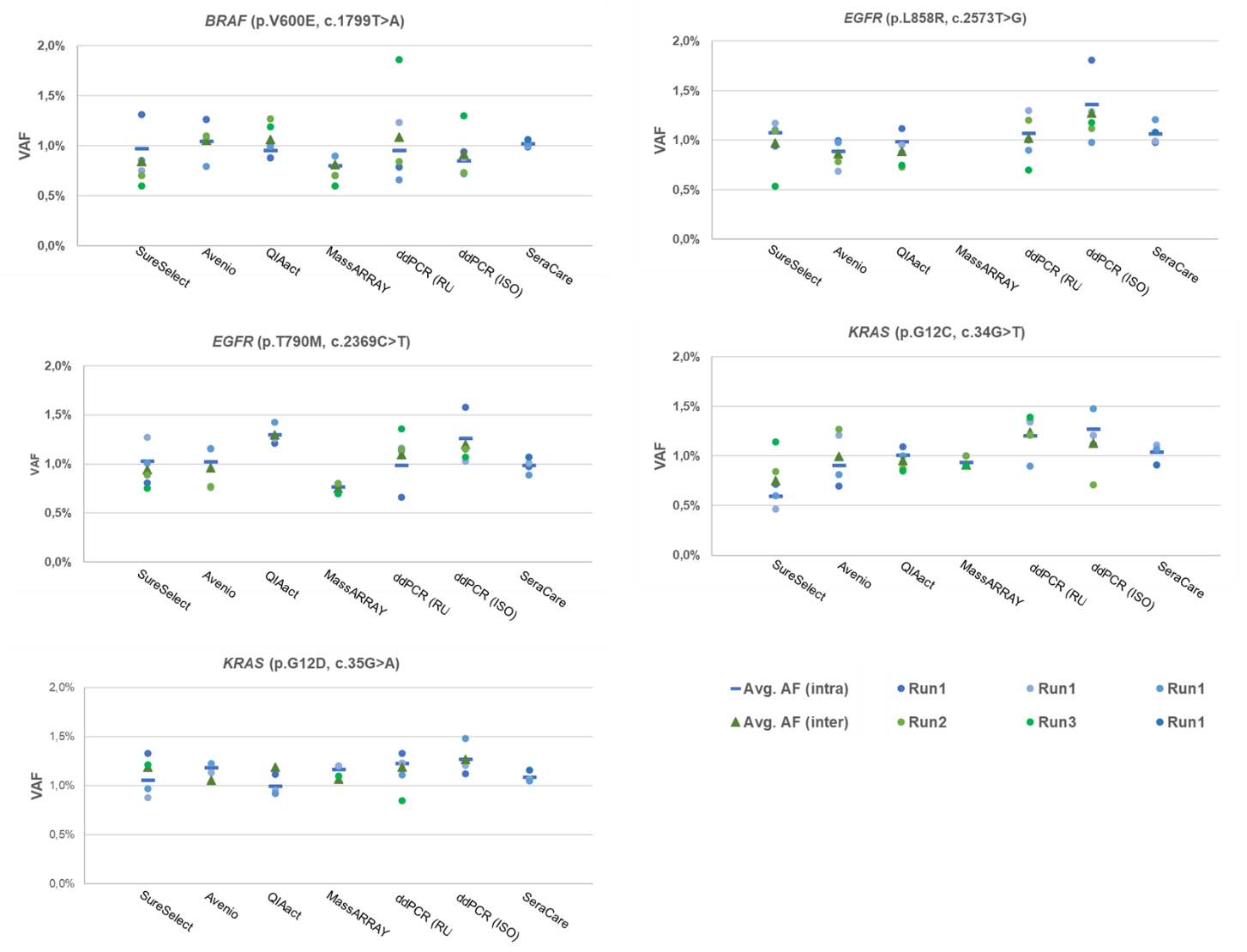


**Figure S1.** Intra-run precision and inter-run reproducibility of three NGS- and three qPCR-based mutation analysis assays. Shown are variant allele frequencies (VAF) of five clinically relevant mutations that are *BRAF* V600E, *EGFR* T790M, *EGFR* L858R, *KRAS* G12C and *KRAS* G12D included in the SeraCare Seraseq ctDNA complete reference material at a VAF of 1%. Variants were assessed from three independent samples preparation sequenced in one run as well as in three separate runs assessed using a customized hybrid capture SureSelect custom panel from Agilent (performed at University of Manchester - UNIMAN), the AVENIO Targeted Assay from Roche, a commercially available hybrid capture based custom panel which employs UMIs, and a commercially available amplicon panel QIAact Lung UMI Panel from QIAGEN (both performed at Medical University of Graz - MUG), two droplet digital PCR (ddPCR) assays (one research use only assay performed at Bayer and one ISO15189 validated assay performed at the University Medical Center Groningen - UMCG) and the UltraSeek lung panel (MassARRAY performed at Agena).


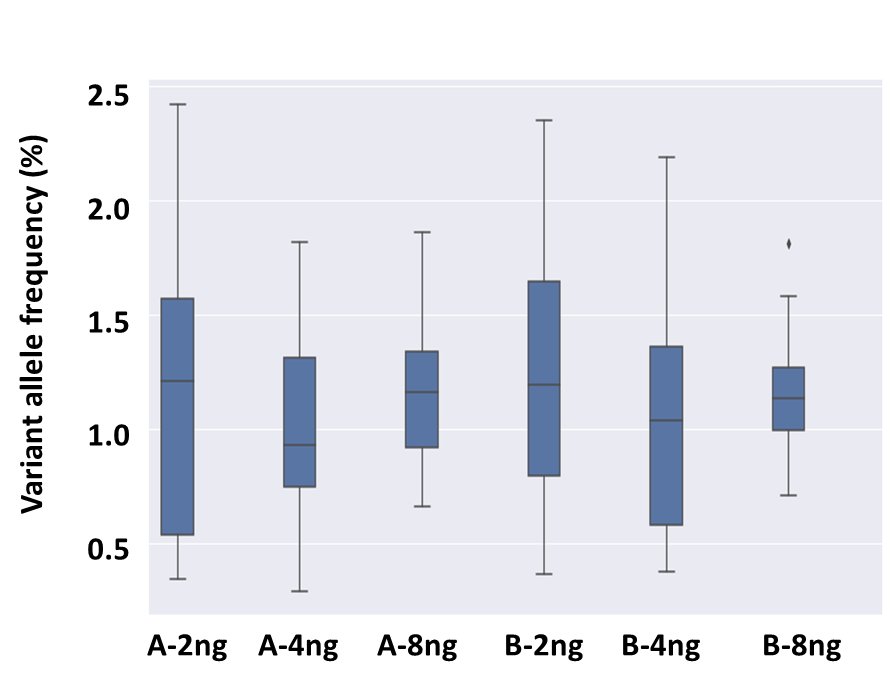


**Figure S2.** Association of input mass quantities and variability in mutation detection of ddPCR. ddPCR of five clinically relevant mutations that are *BRAF* V600E, *EGFR* T790M, *EGFR* L858R, *KRAS* G12C and *KRAS* G12D included in the SeraCare Seraseq ctDNA complete reference material. ddPCR was performed with 2 ng, 4 ng and 8 ng input mass and performed at two sites: (**A**) Bayer and (**B**) UMCG. Lower input amounts resulted in a lower precision of variant calls.


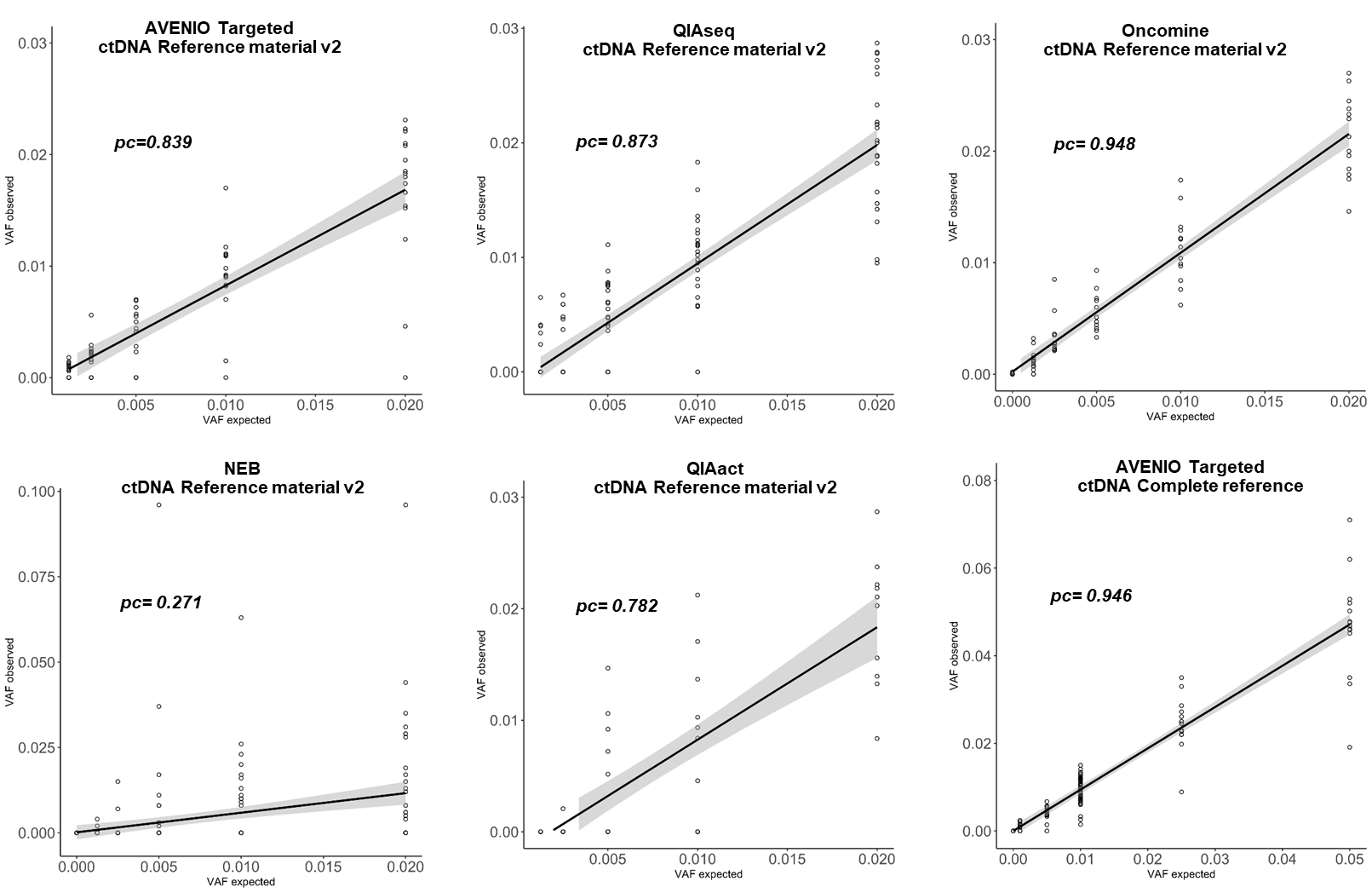


**Figure S3.** Concordance of expected and observed variant allele frequencies (VAF). Linear regression of VAF assessed with the various NGS mutation analysis platforms and the VAF reported from SeraCare. Lin´s concordance correlation coefficient (pc) was calculated.

**
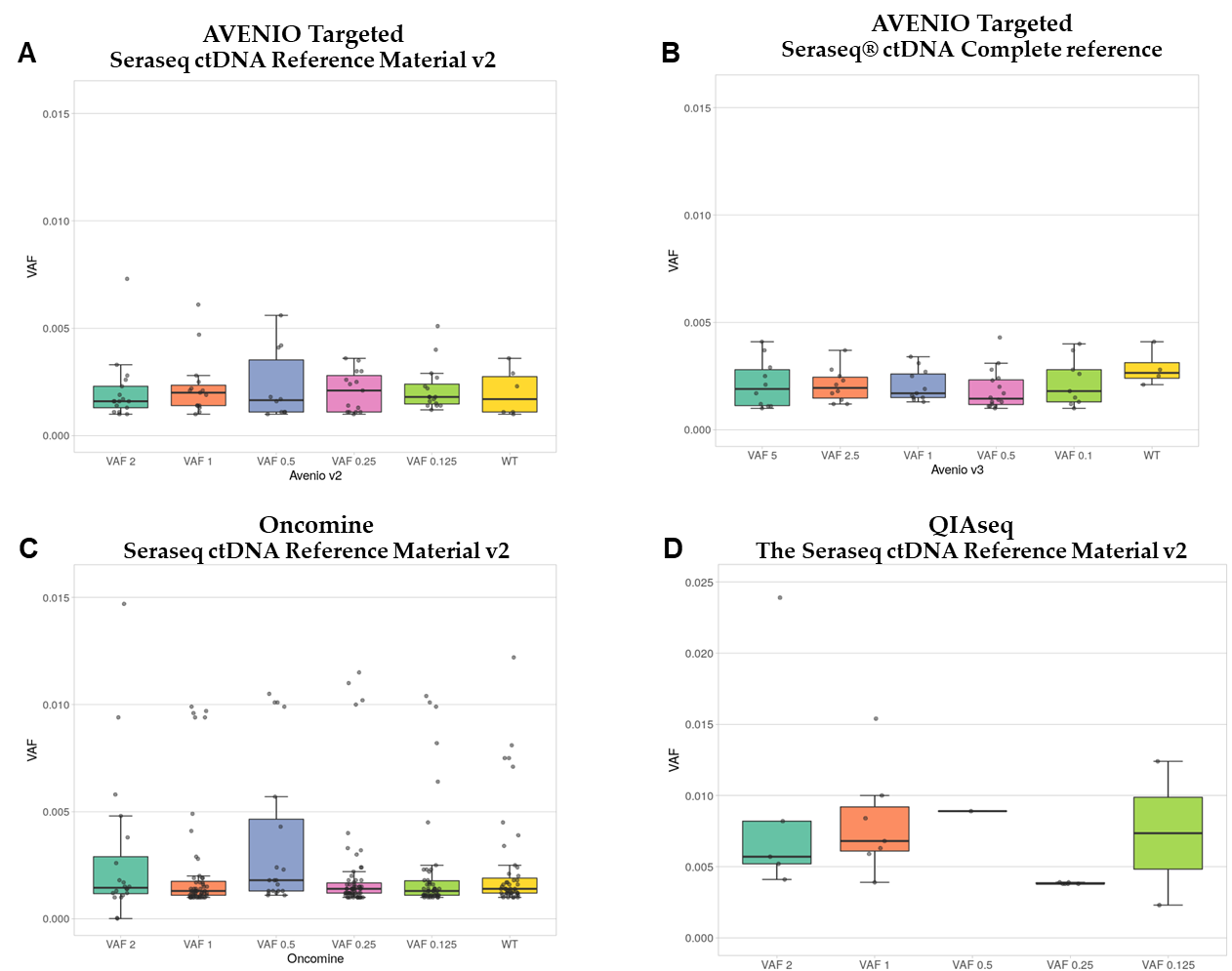
**

**FigureS4.** Boxplots showing the distribution of variant allele frequencies (VAF) of variant calls that are not reported in the reference material. Variants below with VAF <0.1% were excluded. (**A, B**) VAFs of putative false positive (FP) of the AVENIO Targeted kit with two sets of reference material (**C**) For the Oncomine assay high level variants that recurrently occurred due to mispriming were also filtered and are not represented in the plot. (**D**) The QIAseq assay revealed the lowest FP rate, but with substantially higher VAF compared to the other assays.


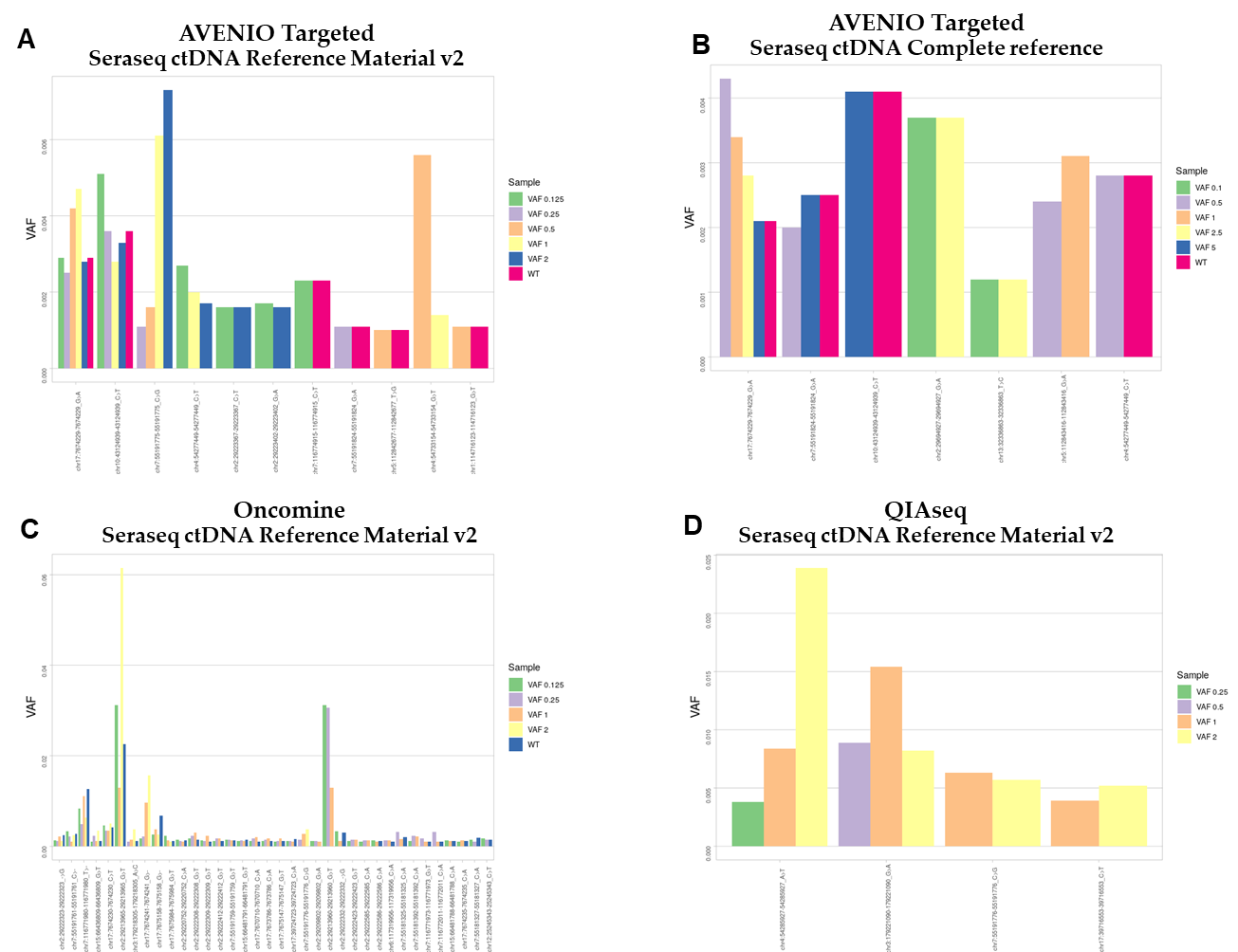


**Figure S5.** Positions with recurrent false positive variant calls. Plotted are putative false positive (FP) variants with their respective variant allele frequency (VAF) that were observed in two or more samples of the reference materials (2%, 1%, 0.5%, 0.25%, 0.125% and WT for Seraseq ctDNA reference material v2 and 5%, 2%, 1%, 0.5%, 0.1% and WT for the Seraseq ctDNA Complete reference). For AVENIO (**A, B**) and QIAseq (**D**) FP calls that occur in two or more samples are plotted. For Oncomine (**C**) only FP that occur in three or more samples are plotted.

**
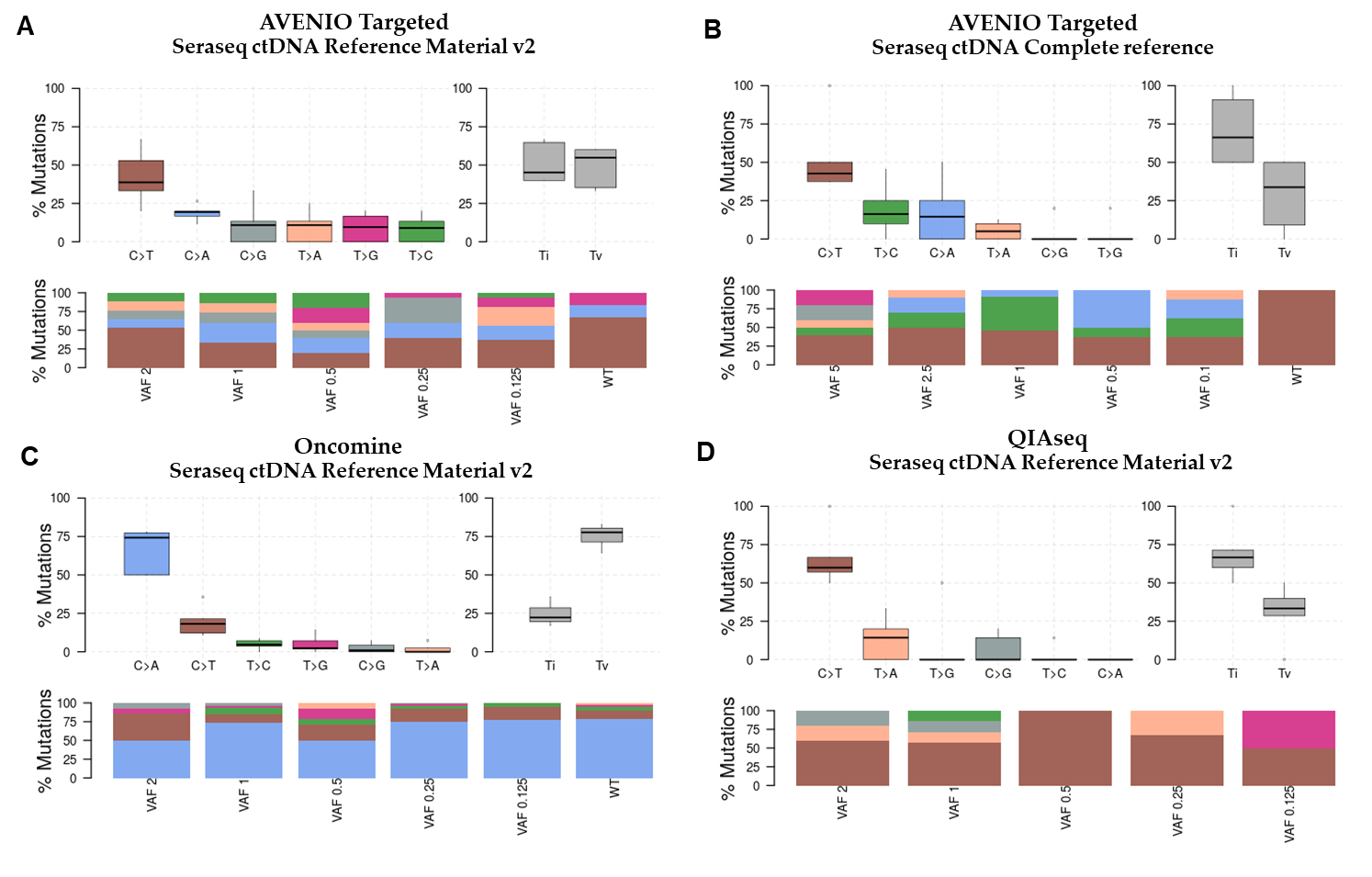
**

**Figure S6.** Summary of false positive (FP) variant calls. **(A–D)** Upper left panels: Boxplots showing the overall distribution of FP variant call across all samples of the reference materials (2%, 1%, 0.5%, 0.25%, 0.125% and WT for Seraseq ctDNA reference material v2 and 5%, 2%, 1%, 0.5%, 0.1% and WT for the Seraseq ctDNA Complete reference) separated per variant class. While for the AVENIO Targeted kit (**A,B**) and the QIAseq assay (**D**) C>T transition were most commonly observed, most FP calls for the Oncomine assays were C>A transversion, which is aloo reflected in the Transition/transversion (Ti/Tv) ratios (upper right panel). Stacked barplots show the fraction of conversions for each reference material (lower panel).


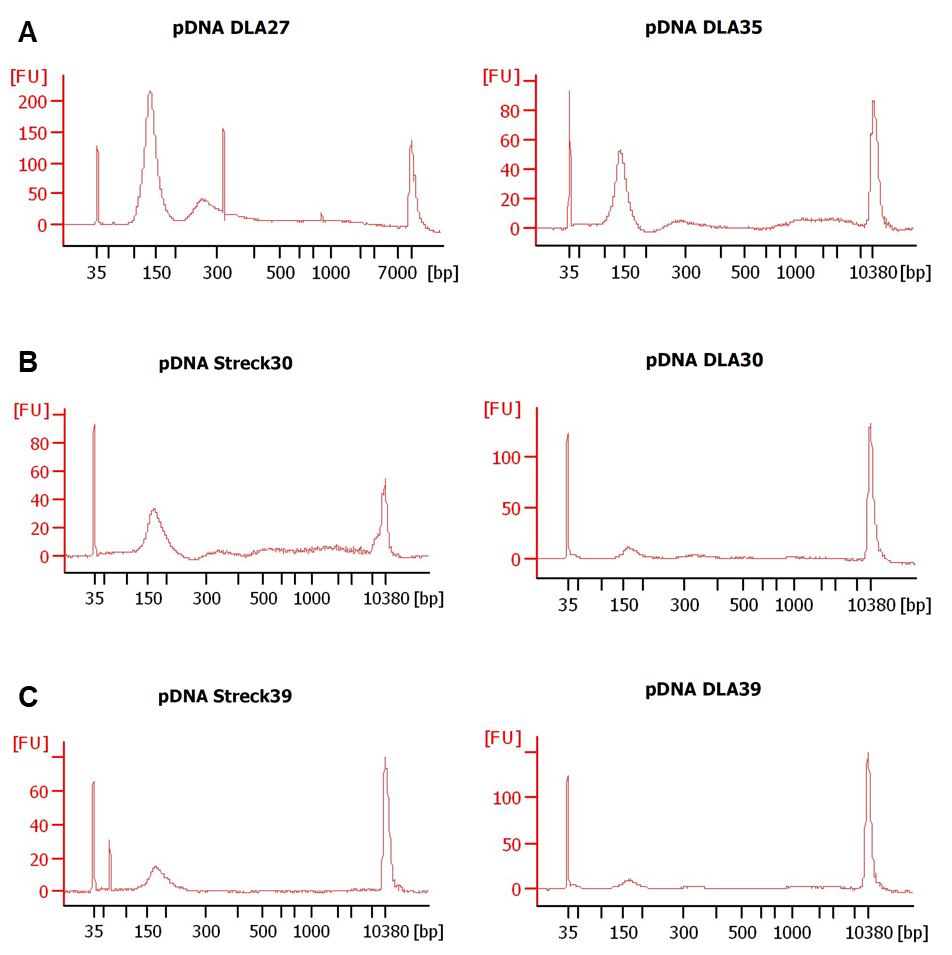


**Figure S7**. Size distribution of cfDNA extracted from Streck and DLA plasma. Shown are a Bioanalyzer traces of (A) DLA samples from two NSCLC patients. The leftmost and rightmost peaks (labeled 43 and 113) are size markers of 35 bp and 10380 bp, respectively. The average length of the cfDNA is calculated to be 160 bp, corresponding to mononucleosomal DNA. In addition a minor dinucleosomal peaks at 320bp can be observed. (B), (C) Comparison of cfDNA extracted from Streck and DLA plasma from the same patient. No major difference could be observed with respect to fragment sizes.

Supplementary References

1. Newman AM, Bratman SV, To J, Wynne JF, Eclov NC, Modlin LA, et al. An ultrasensitive method for quantitating circulating tumor DNA with broad patient coverage. Nature medicine. 2014;20(5):548-554.

2. Newman AM, Lovejoy AF, Klass DM, Kurtz DM, Chabon JJ, Scherer F, et al. Integrated digital error suppression for improved detection of circulating tumor DNA. Nat Biotechnol. 2016;34(5):547-55.

3. Xu C, Nezami Ranjbar MR, Wu Z, DiCarlo J, Wang Y. Detecting very low allele fraction variants using targeted DNA sequencing and a novel molecular barcode-aware variant caller. BMC genomics. 2017;18(1):5.
